# Supplementary figures and images for: Exercise Training Impacts Cardiac Mitochondrial Proteome Remodeling in Murine Urothelial Carcinoma
Source: Int J Mol Sci. 2018 Dec 31;20(1):127. doi: 10.3390/ijms20010127 (PMC6337197; doi:10.3390/ijms20010127)

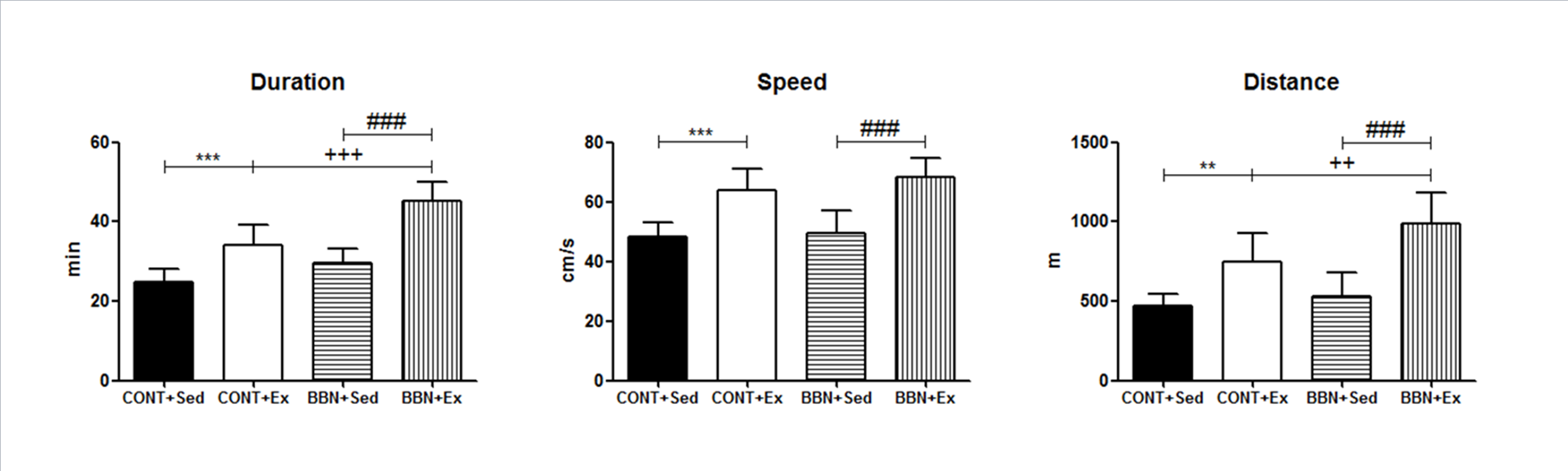

Supplement: Supplementary file 1 [file ijms-20-00127-s001.zip › Supplementary Information/Supplementary Figure S1.tif]

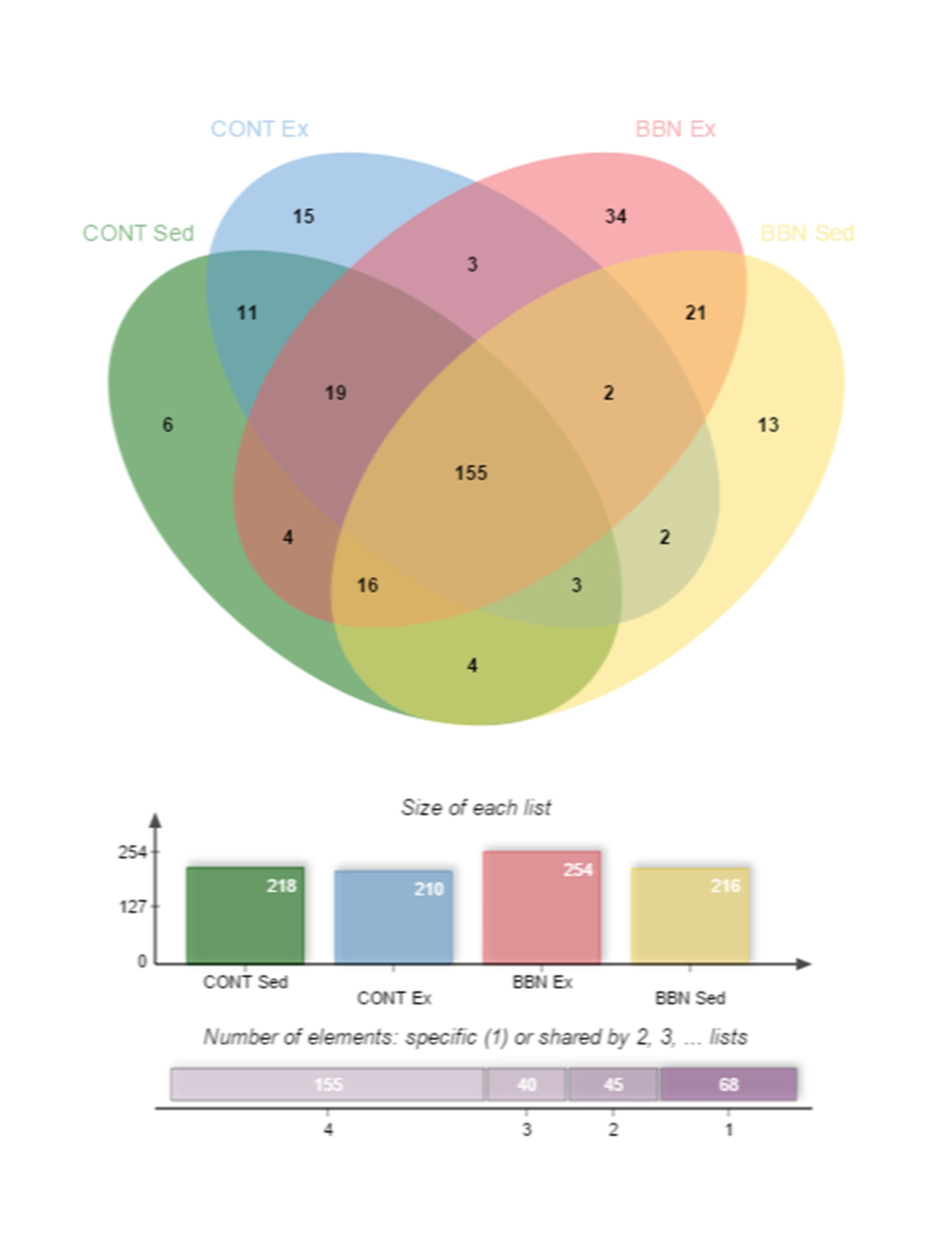

Supplement: Supplementary file 1 [file ijms-20-00127-s001.zip › Supplementary Information/Supplementary Figure S2.tif]
